# Supplementary material for: An uncertainty-based model of the effects of fixation on choice
Source: PLoS Comput Biol. 2021 Aug 16;17(8):e1009190. doi: 10.1371/journal.pcbi.1009190 (PMC8389845; doi:10.1371/journal.pcbi.1009190)
Supplement: S1 Text — (PDF) [file pcbi.1009190.s001.pdf]

S1: PUC model variants

We considered three variants of the main PUC model:

- In the "flexible prior variance" model, the prior variance parameter σ_p^2 is a free parameter.
- In the "flexible prior mean & variance" model, both the prior mean μ_p and the prior variance are free parameters.
- In the "uncertainty-neutral" model, the uncertainty aversion parameter A is set to zero, meaning that the utility of an item is equal to the posterior mean of the item's value.

We compared these models to the main PUC model in terms of their log likelihood, AICc, and BIC (Table A). We found that the main PUC model performed best. The uncertainty-neutral model performed worse than the main model, indicating that the uncertainty aversion term helps to explain people's choices.

To check for potential heterogeneity in the population, we conducted hierarchical Bayesian model selection [1] on the main PUC model and its three variants, using the VBA package [2]. This analysis indicated that by far the largest proportion of the population follows the main PUC model (97.4% based on AICc and 94.9% based on BIC).

	flexible prior var	flexible prior mean & var	uncertainty-neutral
neg LL	19 (34, 9)	49 (71, 33)	-655 (-455, -923)
AICc	-56 (-75, -25)	-92 (-124, -47)	-1219 (-1756, -835)
BIC	-140 (-159, -108)	-256 (-288, -212)	-1133 (-1666, -741)

Table A. Comparison between the main PUC model and its variants, in terms of differences in negative log likelihood, AICc, and BIC. Negative values mean that the main PUC model is better. (Of course, the log likelihood of a more flexible model will always be higher.) All values are summed across subjects, with bootstrapped 95% confidence intervals given in parentheses.

References

1. Stephan KE, Penny WD, Daunizeau J, Moran RJ, Friston KJ. Bayesian model selection for group studies. NeuroImage. 2009;46(4):1004–1017.

2. Daunizeau J, Adam V, Rigoux L. VBA: a probabilistic treatment of nonlinear models for neurobiological and behavioural data. PLoS computational biology. 2014;10(1).
