# Supplementary material for: An uncertainty-based model of the effects of fixation on choice
Source: PLoS Comput Biol. 2021 Aug 16;17(8):e1009190. doi: 10.1371/journal.pcbi.1009190 (PMC8389845; doi:10.1371/journal.pcbi.1009190)
Supplement: S2 Text — (PDF) [file pcbi.1009190.s002.pdf]

S2: Details of model fitting

Parameter ranges

In parameter fitting using Bayesian Adaptive Direct Search, one has to set a range for each parameter that is fitted. A range that is too small may cause the true optimum to fall outside the range. A range that is too large may slow down the optimization or increase the risk of local optima. We set the parameter ranges by trial and error, always ensuring that the fitted parameters did not reach the bounds of the range.

The parameter ranges in the PUC model were as follows:

- The measurement variance σ^2 is always positive. We assigned an upper bound of 900 and a lower bound of e^{-10}.
- For the uncertainty aversion parameter A, we chose a range from -10 to 32.
- The collapsing bound parameters B_0, λ, and k were all restricted to be positive, and we gave them a lower bound of e^{-10}. We choose upper bounds of 100 for B_0 and λ and 20 for k.
- The guessing rate g is a probability and therefore between 0 to 1. In the Weibull function, we used parameter values $a = 21.14$ and $b = 1.38$ in the *wblcdf* function in MATLAB.
- We set non-decision time τ to 0, 100, 200 or 300 ms, given that the temporal resolution of the fixation data that we fitted was 100 ms.

The parameter ranges in the aDDM were as follows:

- All parameters had a lower bound of e^{-10}.
- We gave the scaling constant d an upper bound of 0.1.
- Instead of σ directly, we fitted the scaled quantity $\mu = \frac{d}{\sigma}$, which we assigned an upper bound of 150.
- The attentional bias parameter θ had a upper bound of 1.5.
- The guessing rate and non-decision time parameters had the same ranges as in the PUC model.

For the parameters in the acbDDM that are shared with the aDDM, we used the same parameter ranges as in the aDDM. For the boundary parameters in the acbDDM, we gave k an upper bound of 20 and λ an upper bound of 2000, with a lower bound of e^{-10} for both.

Multi-start

When fitting parameters, it is typically a good idea to try multiple random starting points, in order to increase the chance of finding the global optimum. But how to choose the number of starting points? For a somewhat informed choice of this number, we estimated the regret of our fit using a method suggested by Acerbi et al. (see supplement of [1]):

1. Regret is a function of two positive integers M and N, with $M < N$.
2. Choose N random starting points and run the parameter optimization for each of them. This produces N log likelihood values.

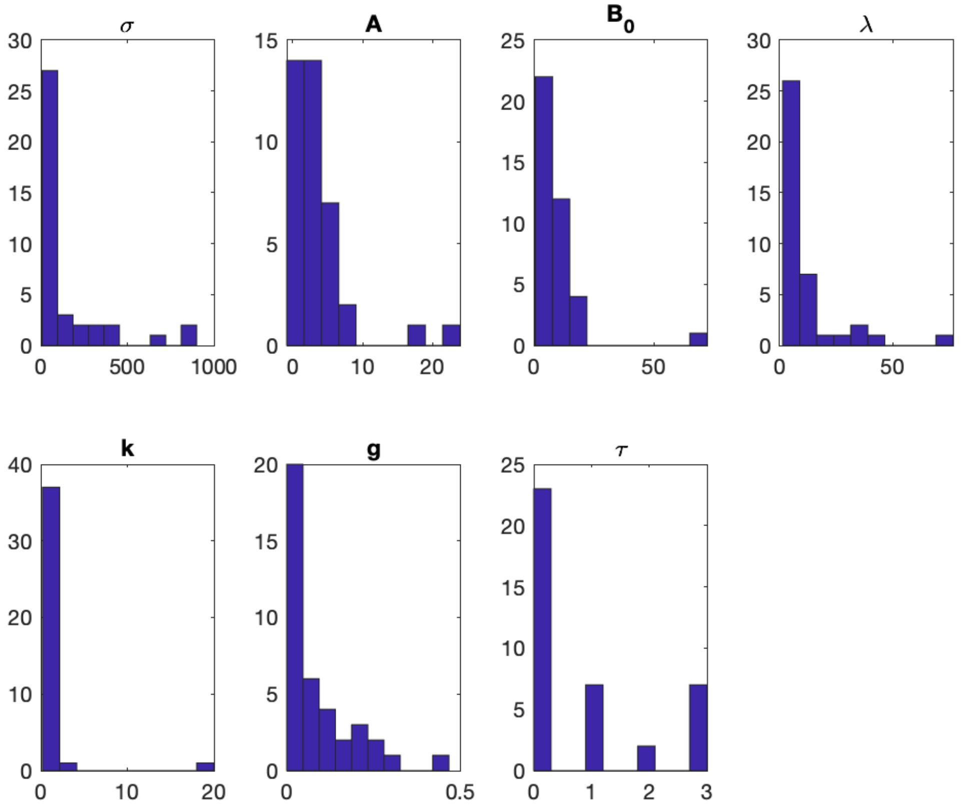

Figure A. Distribution of parameter estimates in the PUC model. σ is the standard deviation of the measurement noise; A is the uncertainty aversion parameter; B_0, λ and k parameterize the collapsing bound function; g is the guessing rate; and τ is the non-decision time.

3. Randomly sample, with replacement, M of these N values and calculate their maximum. This simulates the log likelihood that we would have obtained when using M starting points.
4. To reduce noise, repeat the subset sampling and average the results; we used 50 repetitions.
5. The *regret* associated with the pair (M, N) is the maximum of the N original log likelihood values minus the average of the "subset maximum" log likelihood values.

Finally, we declare a number of starting points N to be *acceptable* if the regret associated with $(N - 10, N)$ is no greater than 1. For example, 50 starting points are acceptable if the regret associated with $(40, 50)$ is no greater than 1. This yields a lowest acceptable number of starting points of 23 for the PUC model, 83 for the aDDM, and 91 for the acbDDM. We used these numbers when fitting these models.

Parameter estimates

In figure A, B and C we show the fitted parameter distribution for the PUC, aDDM and acbDDM models.

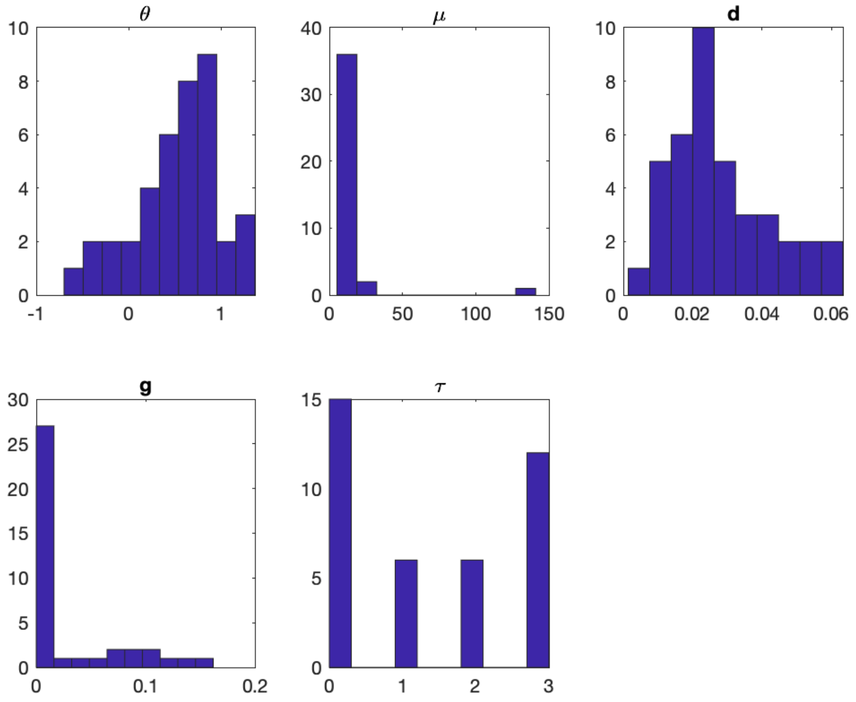

Figure B. Distribution of parameter estimates in the aDDM. θ is the attentional bias factor; μ is the standard deviation of the noise; d is the scaling factor for the decision variable; g is the guessing rate; and τ is the non-decision time.

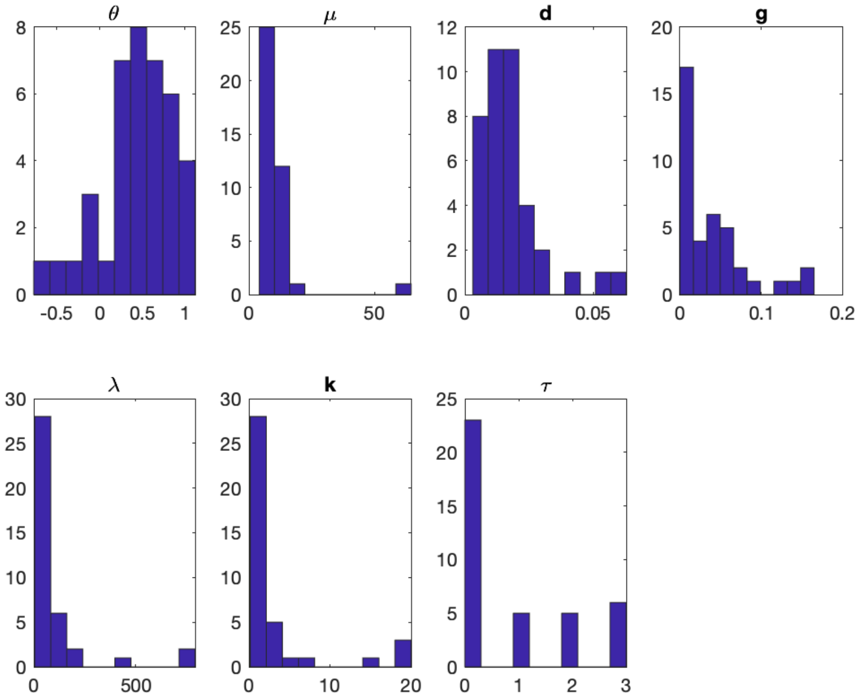

Figure C. Distribution of parameter estimates in the acbDDM. Parameters are as for the aDDM and in addition, λ and k parameterize the collapsing bound.

References

1. Acerbi L, Dokka K, Angelaki DE, Ma WJ. Bayesian comparison of explicit and implicit causal inference strategies in multisensory heading perception. PLoS computational biology. 2018;14(7):e1006110.
