# Supplementary material for: An uncertainty-based model of the effects of fixation on choice
Source: PLoS Comput Biol. 2021 Aug 16;17(8):e1009190. doi: 10.1371/journal.pcbi.1009190 (PMC8389845; doi:10.1371/journal.pcbi.1009190)
Supplement: S3 Text — (PDF) [file pcbi.1009190.s003.pdf]

S3: Parameter recovery

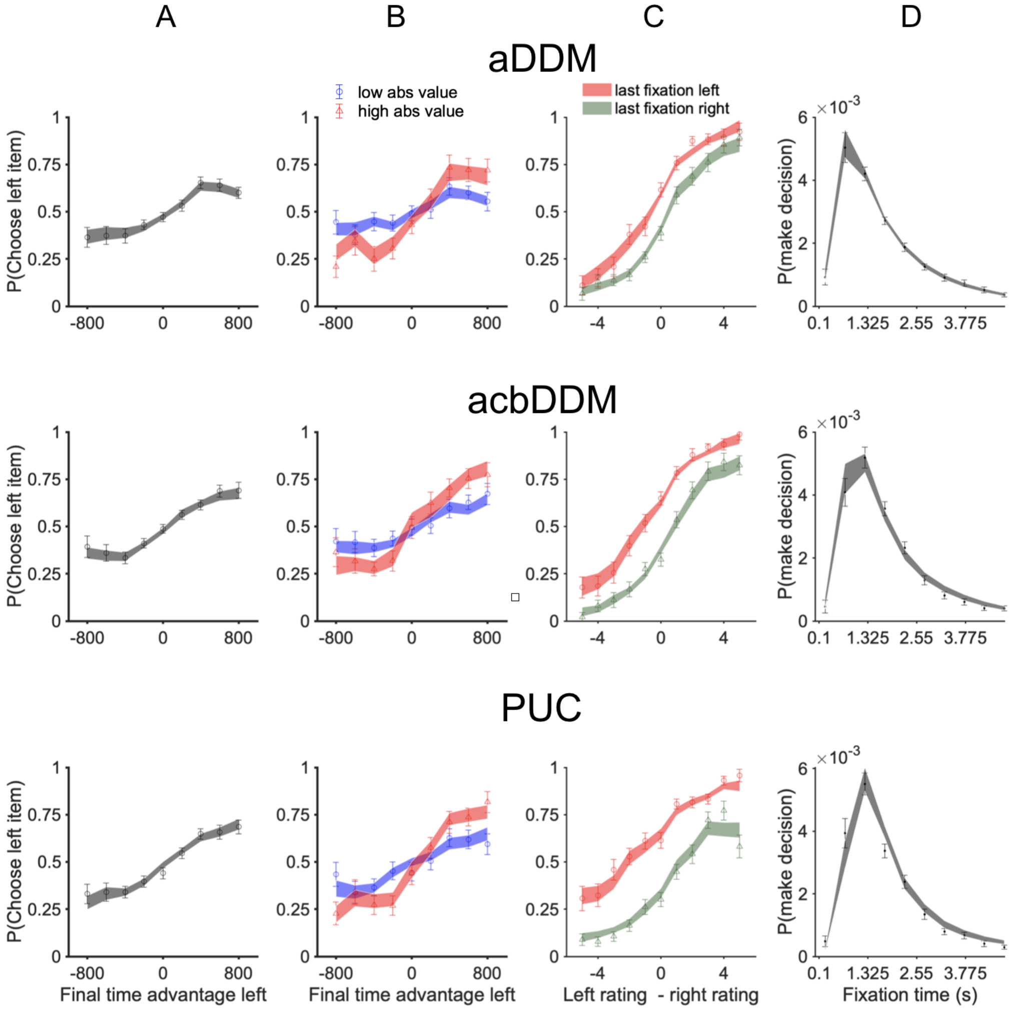

Figure A Fits of the aDDM, acbDDM and the PUC models to summary statistics of synthetic data generated from the same respective models.

Within each model, we performed parameter recovery. We generated and then fitted synthetic data sets using the parameter estimates of the real subjects (for a total of 39 data sets). The model fits to the summary statistics obtained using the fitted parameters were near-perfect (Figure A).

We then considered the parameter estimates themselves. Parameter recovery was good for the aDDM (Figure B), and less good for the more complex acbDDM and PUC models, with some parameters being recovered well and some less well (Figures C and D). This is likely due to trade-offs between parameters, where a change in one parameter can be compensated for by changes in one or more other parameters, to produce an approximately equally high log likelihood. Importantly, however, the results of our paper do not rely on parameter estimates but only on maximal log likelihoods (and AICc/BIC), so issues with parameter recovery will not affect our conclusions.

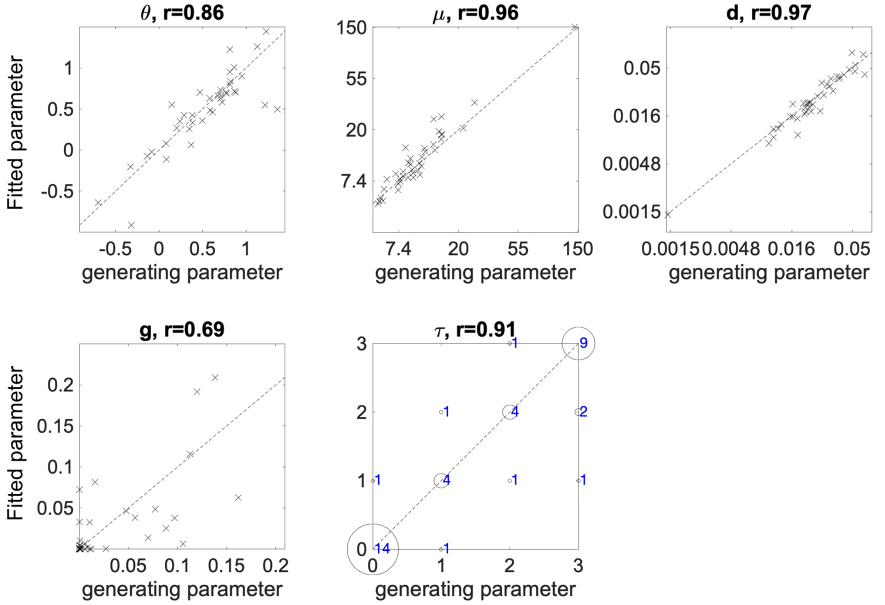

Figure B. Parameter recovery in the aDDM. Shown are the parameter estimates as a function of the generating parameters, with the parameter name and Pearson correlation given in the plot title. Some parameters are plotted in log space because that is how we fitted them; the Pearson correlation is then also calculated for the log parameter. For the non-decision time parameter τ, which has discrete values, circle circumference is proportional to the number of data points as annotated.

References

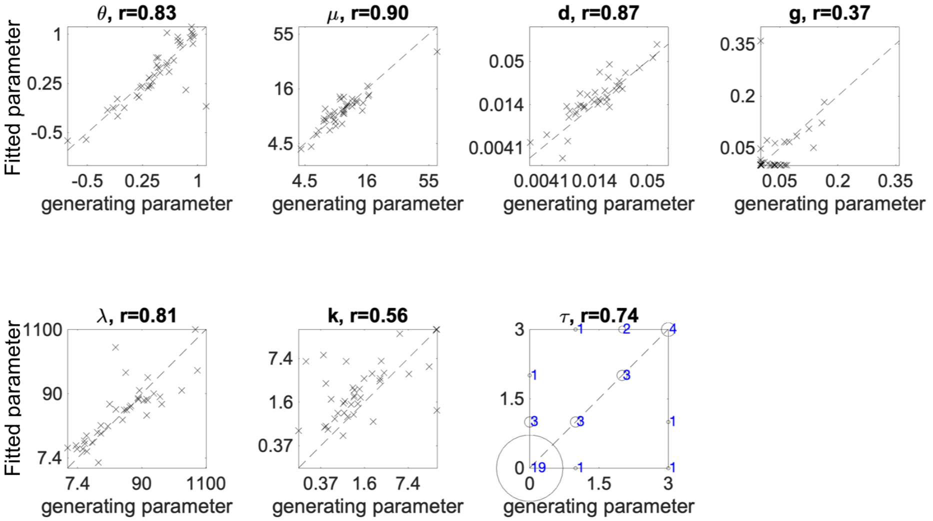

Figure C. Parameter recovery in the acbDDM. For details, see Figure B.

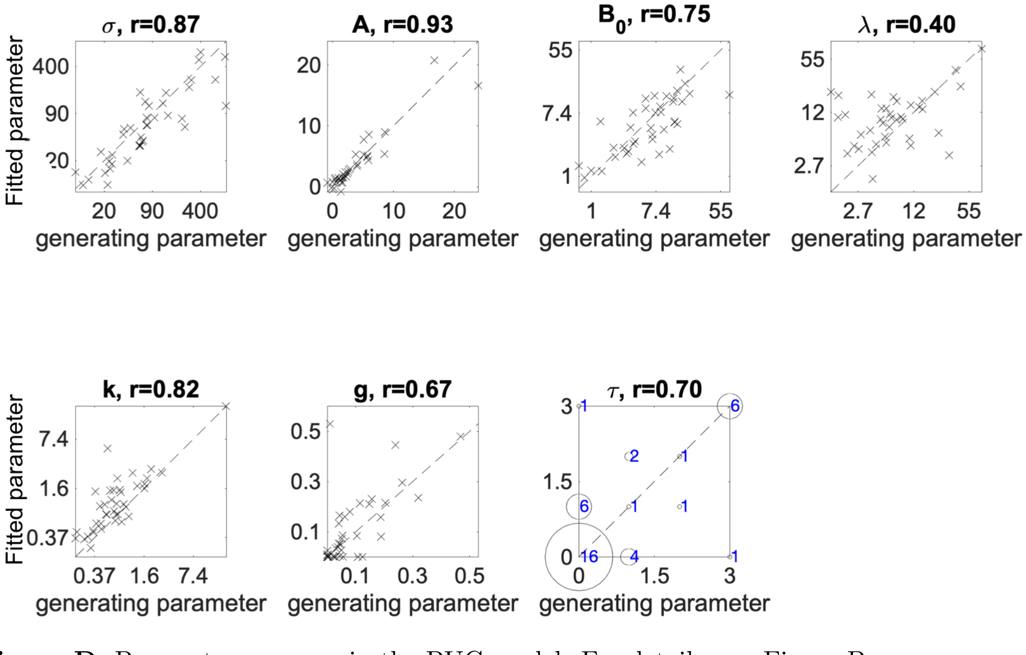

Figure D. Parameter recovery in the PUC model. For details, see Figure B.
