# Supplementary material for: An uncertainty-based model of the effects of fixation on choice
Source: PLoS Comput Biol. 2021 Aug 16;17(8):e1009190. doi: 10.1371/journal.pcbi.1009190 (PMC8389845; doi:10.1371/journal.pcbi.1009190)
Supplement: Attachment — Submitted filename: response_to_review_fixation.pdf [file pcbi.1009190.s004.pdf]

Zhi-Wei Li
6 Washington Place
New York University
New York, NY, 10003
Email: zhiwei.li@nyu.edu

April 26, 2021

Dear editors,

We would like to thank the reviewers for their careful reading our paper and for their valuable comments. We have addressed their comments point by point below. Our responses to the comments are numbered and marked in **blue**. References to updated passages in the manuscript are marked in **green**.

This revision has helped us to improve the paper, and we are very grateful for having been given the opportunity to do so. We hope that the revised version of the manuscript is considered fit for publication in *PLOS Computational Biology*.

Sincerely,

Zhi-Wei Li and Wei Ji Ma

Referee 1's comments: This is a really nice paper investigating a Bayesian approach to value-based decision-making. Drift-diffusion models (DDM) assume that evidence is accumulated over time for each alternative. The attentional DDM (aDDM) assumes that the rate of evidence accumulation increases for the alternative that is attended to. This leads to a choice bias in favor of attended items. This paper proposes an interesting alternative model for this phenomenon. The model assumes that subjects have a prior expected value (and associated variance) for each alternative. When the subject attends to an alternative, they update its expected value and reduce the variance around that expectation. Subjects are risk-averse and so they prefer alternatives that they are more certain about. The preference for higher values and higher certainty leads subjects to choose alternatives that are higher valued but also attended to longer. The model presented in this paper is very sensible and has a lot of nice features. It blends economic intuition about decision-making under uncertainty with cognitive knowledge about attention and information gathering. While the model does not substantially outperform the existing model (aDDM) in goodness-of-fit, it is still an important step, for at least a couple of reasons. First, the aDDM is silent about how/why decision-makers sample information. The authors' PUC model makes it clear that the point of attending to an alternative is to get a more precise and accurate estimate of the value of that alternative. This intuition is critical to advance our understanding of how people sample information. While this paper does not attempt to model the fixation process, one can see how you would use this framework to tackle that question. Second, the authors' model seems to (marginally) outperform the standard aDDM. Only when the authors give the aDDM collapsing bounds do the models perform similarly well. Thus, another takeaway point of the paper is that collapsing bounds are an important feature to consider in these model-comparison exercises, and they may be an important addition to the aDDM (though visually the improvements were imperceptible, see Fig. 3). In summary, I think this is an important and well written paper. I have a number of mostly minor comments. If the authors can address those comments, I would be happy to endorse publication.

(1) Regarding the comparison between the aDDM and cbaDDM: Figure 3 shows little difference between the models. These are all choice plots, where collapsing bounds will have little impact. It might be useful to add a fourth column comparing the reaction time curves with each model compared to the data.

Response 1: We greatly appreciate the positive comments! It is a good idea to compare the model to the data in terms of reaction times. We set out to conduct this analysis. A complication was that a starting point of our modeling approach has been to rely on empirical fixations, and therefore the models cannot predict reaction times that extend beyond the subject's last fixation time. Therefore, for the purpose of these plots, we decided to create synthetic fixation series by drawing from the empirical distribution. This method was introduced by Krajbich et al. 2010 and we previously already used it when doing model recovery. In this approach, we plot the summary statistics of probability of making choice against the total fixation time. We used the distribution of fixation times split out by subject, then independently and sequentially drew from this distribution to create a synthetic fixation series for each trial after the empirical fixation series has ended; we repeated this 10 times for each trial which is enough to achieve a stable result. This allows us to obtain an unrestricted model prediction for total fixation times (which we use as a proxy for reaction times throughout the paper). We report the results of this analysis in the fourth column of Fig. 3 and the accompanying text. The figure now looks as follows:

(2) In comparing the advantages of the PUC to the aDDM, the authors repeatedly state that in the aDDM the "meaning of the decision variable is unclear". I'm not really sure what the authors mean by

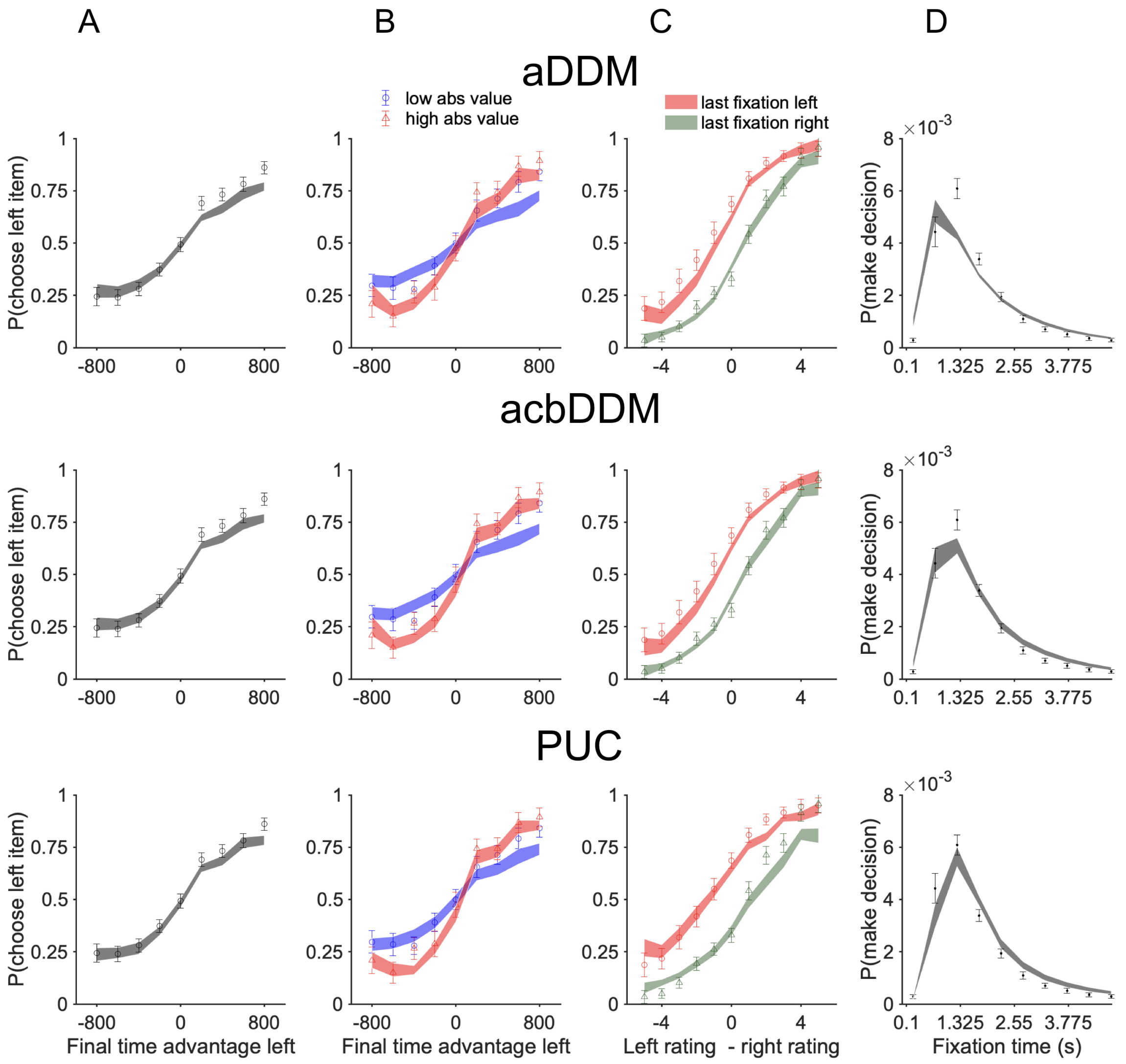

Figure 1: Fits of the aDDM, the acbDDM, and the PUC model to summary statistics of the data. (A) When the total fixation time advantage of an item increases, that item is chosen more often. (B) Same as A but conditioned on item rating. Both models predicted that when the absolute values of both items are higher (i.e. both are more preferred items), the fixation modulation effect is larger, which trend is less significant in the empirical data. (C) Besides total fixation duration, the last fixation also biases the choice. (D) Distribution of total fixation time. Note for this figure we simulated fixation series extending after the empirical total fixation time. The aDDM fits we obtained differ from those in Krajbich et al.(2010) not only because of the differences in parameter estimation methods (see section "Differences from Krajbich et al. (2010)."), but also because of a difference in trial aggregation: Krajbich et al. aggregated the model predictions across rating pairs without taking into account the frequencies of these pairs in the experiment. We instead aggregated individual-trial predictions; this is necessary in our case because we used the individual-trial fixation series, but it also ensures a proportional representation of each rating pair in the summary statistics.

this. In the (a)DDM, the goal is to choose the better item. That is why drift rate is driven primarily by the alternatives' values; this leads to the decision-maker choosing the higher-value item in most cases. As outlined by Webb (2019) in Management Science, the DDM is essentially just a choice rule that takes utilities as inputs and outputs logit choices. In other words, the authors' model first updates values then calculates utilities, while the DDM first generates utilities then updates the decision variable. It isn't that one model is more normative than the other, its that the models do things in a different order.

(3) More generally, I think that the authors claims about what is normative (or what isn't) should be set aside. What is normative depends on lots of assumptions. The aDDM could certainly be normative if attention switching is costly and attention is limited; we know that the standard DDM is normative under certain conditions (Wald,1945). The authors' model is already admittedly semi-normative, and that doesn't even consider the fact that risk-aversion is not normative per se (though it is standard) and that there is no explanation in this model for the fixation series themselves, which seemingly lead to non-normative information gathering. I would instead focus on the fact that this model is explicitly Bayesian and so is set up to tackle important questions of how attention should be allocated, how to incorporate prior information about the alternatives, what confidence ratings the model should produce, etc.

Response 2: Thank you and we agree! In response to both comments, we have changed our wording to state that the model is explicitly Bayesian, instead of making broad claims about normativity. Specifically, in the abstract, we now write:

The leading model of this effect is a drift-diffusion model with a fixation-based attentional bias. Here, alternatively, we propose an explicitly Bayesian account for the same data.

In the section "Differences between PUC model and aDDM" section", we now write:

the PUC agent chooses the item with the highest utility, whereas the a(cb)DDM does not have an immediate interpretation in terms of utility. (This stands in contrast to the basic DDM model, in which the decision variable can be interpreted as the difference between the values of the two items (Webb et al. 2019)).

Finally, in the Discussion, we now write:

The PUC model postulates what the agent cares about at a behavioral level, through a utility function derived from a posterior distribution over value. By contrast, the a(cb)DDM is neither stated in terms of utility nor involves computing a belief over value.

(4) The authors mention at some point that, unlike in the aDDM, later evidence has a smaller effect on the decision variable. Could the authors elaborate on this point? In Bayesian models the order of data is not supposed to matter. Is that violated here? Or do the authors simply mean that there are diminishing returns to information collection from the same alternative?

Response 3: Yes, the latter. We realize that this was not stated clearly. We now clarified in the manuscript as follows:

In the PUC model, later measurements have a smaller effect on the decision variable than earlier ones,

because all measurements are generated from the same distribution and there are diminishing returns to information collection as the estimated value approaches the underlying true value. By contrast, in the a(cb)DDM, the variance of the noise added at each time point stays the same across time.

(5) In simulating their model, the authors use 100ms time steps, which is much larger than other papers in the literature. Did the authors verify that such large time steps do not lead to distortions in the simulated data? In particular, did the authors do any parameter recovery exercises to ensure that this method was effective?

Response 4: Yes, we performed parameter recovery but did not yet incorporate that in the original submission. We did generate synthetic data by using the exact same rating distribution as the real data, fitted the synthetic data with the same procedure as in fitting real data (using Bayesian Adaptive Direct Search to find the fitted parameters), then compared the resulting model predictions with the synthetic data. In Fig. 2, we confirmed that the summary statistics can be near perfectly recovered with all the models. In addition, Fig. 3 for the aDDM and Fig. 4 for the PUC model (in its simplified form – see response to point (7) below), we show that parameters can be decently well recovered in both models. More complex models like PUC and acbDDM (Fig. 5 do show less good of a parameter recovery. Note, however, that our paper does rely on parameter estimates but only on model comparison.

A finer time step is not necessarily better, because we (like everyone else) assume independent noise across the time points in the model. When the time step approaches the autocorrelation time of single neurons (1-10 ms), then this assumption will be less justified. In addition, changing the time step selection will only change the scaling of drift rate and noise in the aDDM; in the PUC model, the scaling of the observation variance parameter and the boundary time step parameter will change accordingly. Thus, the predicted results will not change in a major way because the change in time step will be compensated for by the re-scaling of these other model parameters.

We have incorporated these parameter recovery results to our main manuscript in the supplement. In the main text we added this method paragraph:

Parameter recovery and model checking. To confirm the validity of our model fitting choices, we fitted synthetic data using the same fitting procedures as for the real data. To generate synthetic data, we used the exact same rating distribution as the real data by matching each synthetic trial with a real trial. Each synthetic subject had different set of parameters randomly chosen from a given range. Then we performed fitting for individual synthetic subjects using methods introduced above. Results are presented in the supplementary material **??**. The summary statistics are recovered very well. Parameter recovery is generally good but somewhat worse for the more complex models (acbDDM and PUC). This is likely due to soft trade-offs between parameters. As a result, the parameter estimates in the real data should be taken with a grain of salt. However, the results of our paper do not rely on parameter estimates but only on log likelihoods and summary statistics. Therefore, the results are not affected by issues with parameter recovery.

(6) On a related note, in their simulations the authors use the empirical fixation series. What happened when the observed fixation series terminated (in the data) and a decision hadn't yet been reached in the simulation? Did the decision suddenly end or were additional fixations generated in some way?

Response 5: Thank you, we have clarified in the main text as follows:

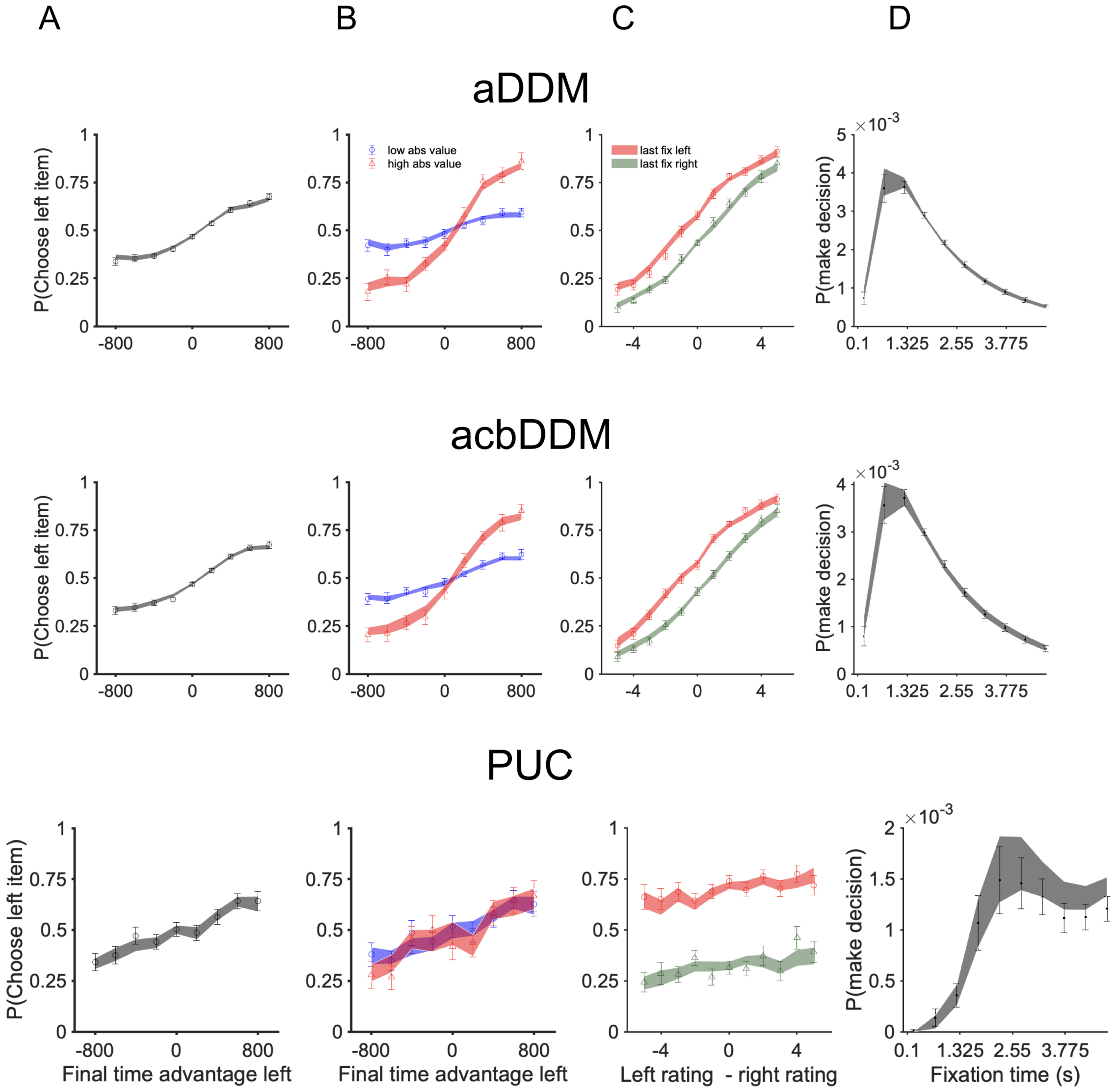

Figure 2: Parameter recovery with synthetic data. The fitted parameters are able to perfectly recover the major summary statistics.

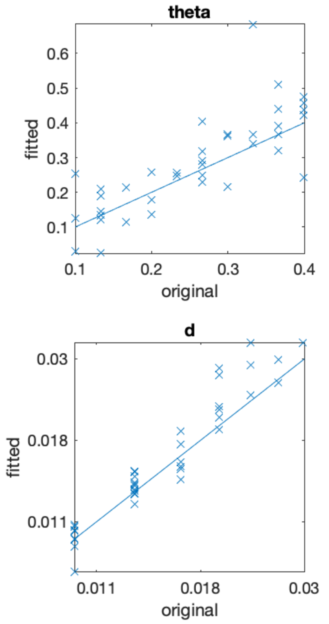

Figure 3: aDDM parameter recovery.

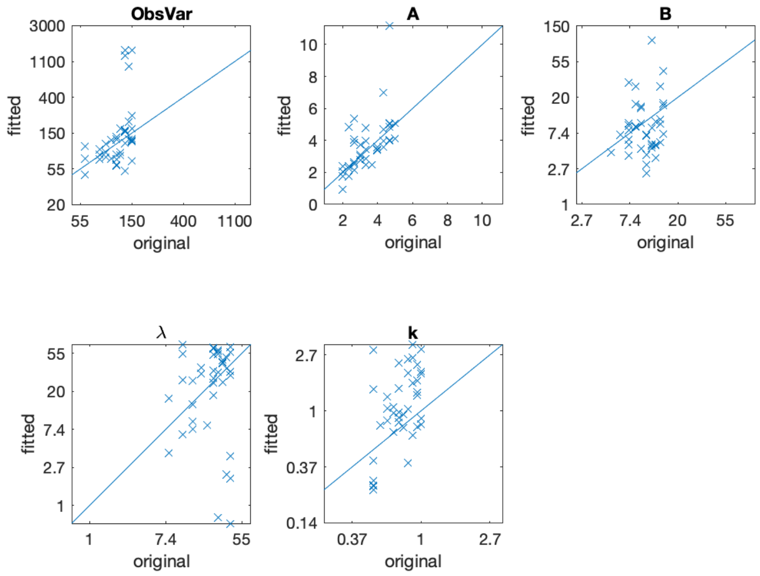

Figure 4: PUC model parameter recovery. The off-diagonal estimates are likely due to some soft trade-offs between parameters.

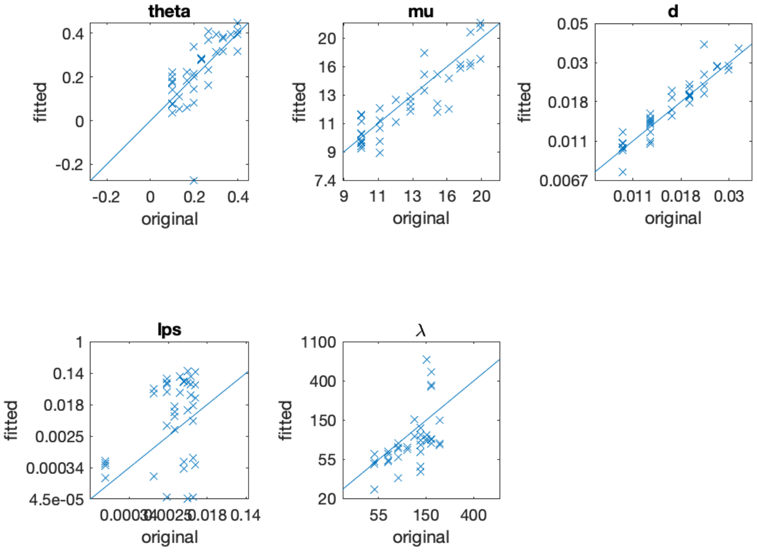

Figure 5: acbDDM model parameter recovery. The off-diagonal estimates are likely due to some soft trade-offs between parameters.

Krajbich et al. used randomly sampled fixation durations from the empirical distribution to perform the simulation both in this and later work. [...] Instead, we used the actual fixation data for each trial, simulating only until the time when the actual fixation has ended and calculating the probability that the choice was made at the end of the empirical fixation series. All remaining probability went into a single bin representing later decision times.

(7) I found the simplified PUCp to be a strange model to consider. Why would subjects have a prior of 0 when only appetitive items were presented in the experiment? Even if subjects for some reason started the experiment with this prior, one would think it would change after the rating task, or at least after a handful of choice trials. Why not instead try a model where the prior mean is the mean of each subject's rating distribution? With a prior of 0, you are essentially guaranteeing that more attention will lead to a higher choice probability. The whole thing seems quite anti-Bayesian. To be clear, I'm not demanding that the authors test such a model, I would just like more explanation why they are even considering this prior=0 model.

What would be worth doing is looking at the relation between the recovered mu_p parameters and subjects' actual mean ratings.

Response 6: Great suggestion! We now adjusted the PUC model so that the prior mean is fixed at each participant's empirical mean and the prior variance is fixed at the empirical variance. In the "Model fitting and model comparison" section in the main text, we added the following:

The PUC model, as introduced in "Decision models" above, has 4 parameters for the value estimation (σ, σ_p, μ_p,A), three bound parameters (B_0, k, and λ), one lapse rate parameter (l) and a non-decision time parameter τ. To simplify, we fixed the prior mean σ_p and variance μ_p to be the empirical mean and variance extracted from the rating data, thus leaving 5 parameters to be fitted. We tested the

more flexible versions of PUC too.

We compared this simplified model with full model and found that it actually performs at least as good as if not better than the full model. So indeed, the actual rating distribution seems to be a good approximation to the model prior. Accordingly, we made the simplified version the main PUC model, and the one in which prior mean and variance are fitted is now a variant. We also fitted the uncertainty-neutral model variant of the simplified model and found that it still fitted worse than the simplified model.

We added these results in the manuscript results, discussion and supplementary materials. The model comparison of the PUC variants are shown in the following table:

	flexible prior var	flexible prior mean and var	uncertainty neutral
neg LL	19 (34, 9)	49 (71, 33)	-655 (-455, -923)
AICc	-56 (-75, -25)	-92 (-124, -47)	-1219 (-1756, -835)
BIC	-140 (-159, -108)	-256 (-288, -212)	-1133 (-1666, -741)

Table 1: Comparison between the main PUC model and its variants, in terms of differences in negative log likelihood, AICc, and BIC. Lower means that the main PUC model is better. All values are summed across subjects; bootstrapped 95% confidence intervals are given in parentheses. The first two columns represent models with more free parameters (prior variance, or both prior mean and prior variance); the third represents a reduced version of the PUC model that does not have an uncertainty-averse term in the utility (i.e. $A = 0$). All PUC model variants fit worse than the main PUC model.

We thank the reviewer for this major improvement.

On that note, it would be useful to see the distributions of recovered parameters for each of the models (in the appendix would be fine).

Response 7: Good point. Now reported in the appendix Figure 4, 5 and 6. We also attach them here:

(8) There has been a debate about the multiplicative vs. additive nature of attention in these choice processes, with Cavanagh et al. 2014 (JEP: General), Smith & Krajbich 2019 (Psych Sci), and Westbrook et al. 2020 (Science). I wonder if the authors could comment on how their findings might inform this debate. Their model would seem to fall somewhere in between, with the uncertainty reduction being “additive” (independent of value), while the benefit of updating from the prior would be “multiplicative”, in the sense that it is more beneficial the farther the true value is from the prior.

Response 8: We agree with this observation! We now remark on this property when comparing PUC with aDDM:

in the PUC model, two main mechanisms influence the preference: the uncertainty reduction term is independent of the item rating, indicating an additive effect of attention, whereas the posterior mean update depends on the difference between the prior mean value and the specific item value, thus indicating a multiplicative effect of attention. In contrast, a(cb)DDM will always boost the item with higher value more, thus the influence of attention is always multiplicative. For more studies regarding the additive versus multiplicative nature of attention, see Cavanagh et al. 2014, Smith et al. 2019 and

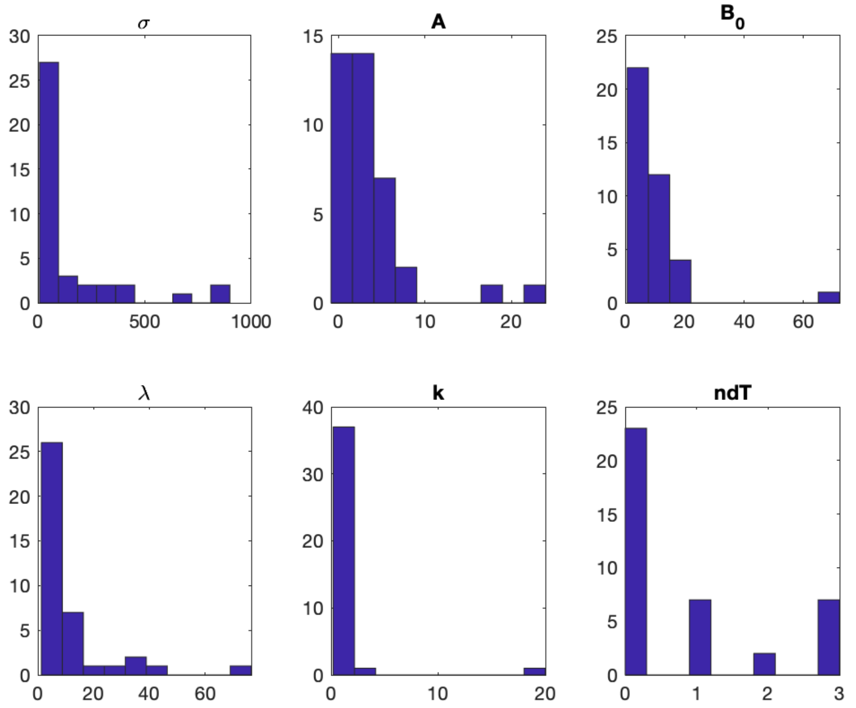

Figure 6: The distribution of fitted parameters in PUC. σ is the variance of sample, A is the uncertain aversion factor, B_0, λ and k are the parameters for the collapsing bound function, l is the lapse rate and ndT is the non-decision time.

Westbrook et al. 2020.

Minor comments (9) There are some references the authors might find useful (in addition to the ones mentioned above). Gwinn & Krajbich 2020 (JESP) - they discuss how certainty (as well as accessibility) about item ratings affects choice outcomes. Parnamets et al. 2015 (PNAS) - they provide evidence for how exogenous manipulations of attention affect choice outcomes Tavares et al. 2017 (Frontiers) - they essentially replicate Parnamets, but with perceptual stimuli

There are also a number of papers on these attention effects that the authors might want to consider including in their references. See Krajbich 2019 (Current Opinion in Psychology) for a review.

Response 9: Thank you, these are all very good references! We have added them in our introduction and discussions.

(10) I'm not sure, but I'm concerned there may be an error in Equation 7. It would seem that as T goes to infinity (as deliberation time increases), $mu_{posterior}$ goes to zero. But perhaps I am missing something.

Response 10: That's right! Corrected now as:

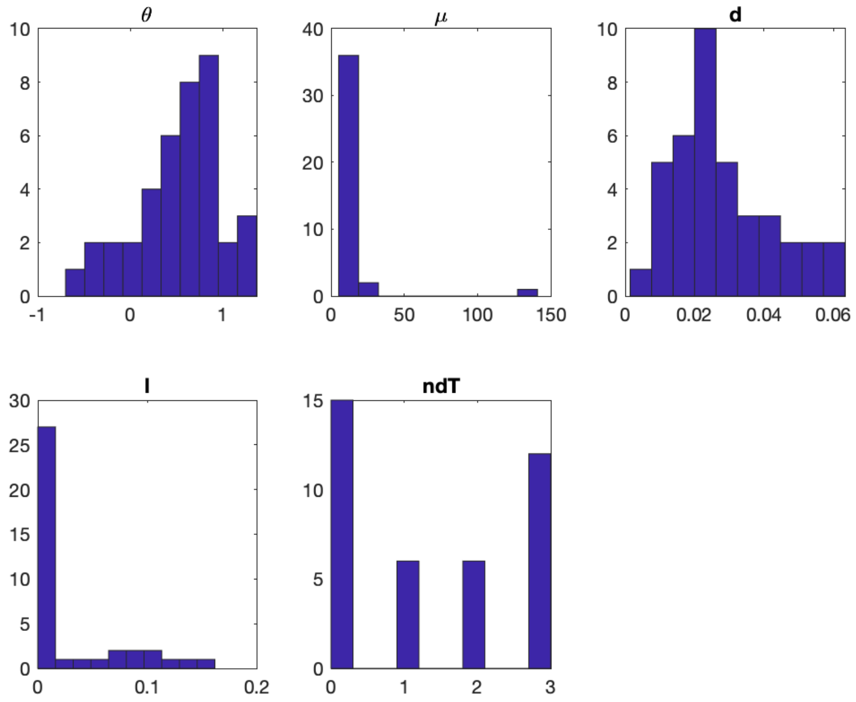

Figure 7: The distribution of fitted parameters in aDDM.θ is the attention bias factor, μ is the noise standard deviation of each step of drifting, d is the scaling factor for the decision variable. l is the lapse rate and ndT is the non-decision time.

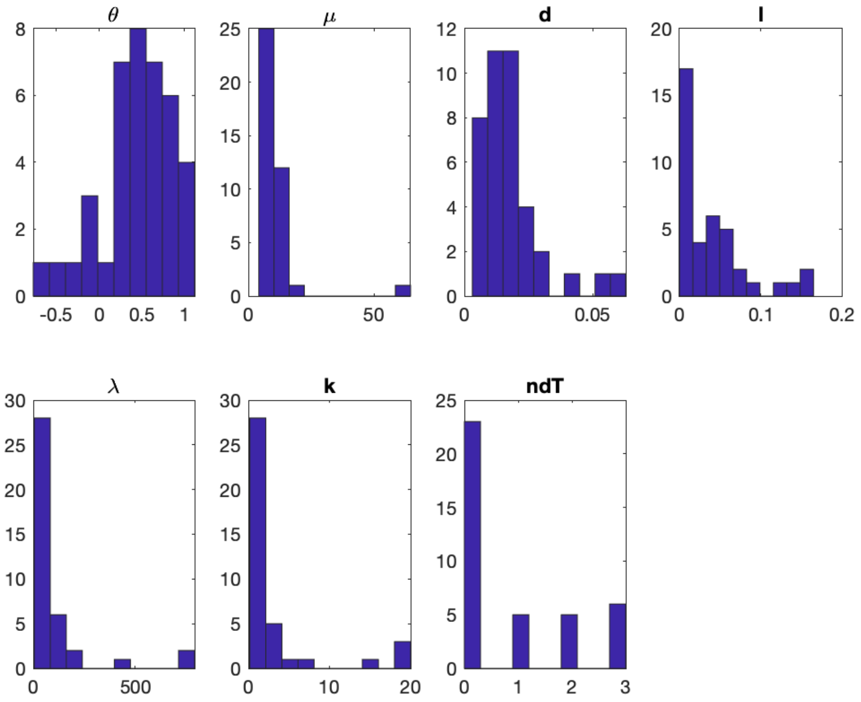

Figure 8: The distribution of fitted parameters in acbDDM. The distribution of fitted parameters in acbDDM. θ is the attention bias factor, μ is the noise standard deviation of each step of drifting, d is the scaling factor for the decision variable. λ and k are the parameters for the collapsing bound. l is the lapse rate and ndT is the non-decision time.

$$\mu_{\text{posterior}} = \frac{\frac{\mu_p}{\sigma_p^2} + \frac{T\bar{x}}{\sigma^2}}{\frac{1}{\sigma_p^2} + \frac{T}{\sigma^2}} \tag{1}$$

$$\sigma_{\text{posterior}} = \frac{1}{\sqrt{\frac{1}{\sigma_p^2} + \frac{T}{\sigma^2}}}, \tag{2}$$

(11) Non-decision time is also thought to account for early orienting to the stimuli, in addition to motor latency at the end of the decision.

Response 11: Indeed we left that out in our modelling for the sake of simplicity.

(12) In the aDDM, d is a scaling constant, not the drift rate per se. The drift is d multiplied by the attention-weighted values.

Response 12: Thanks, corrected as follows:

when fixating on the right item, where d is a scaling constant, r_{left} and r_{right} are the ratings for the two items, and ϵ_t is diffusion noise, drawn independently across time points from a normal distribution $\mathcal{N}(0, \sigma^2)$.

(13) The authors say that in the data the median RT was 4 seconds. Can they elaborate on this, given that in Krajbich et al. 2010, mean RT appears to be about 2 seconds?

Response 13: Thanks, it was a typo and it should be 1.4 seconds. Corrected in the main text.

(14) The authors claim that Krajbich et al. 2010 used a "fixation model" in their simulations, but in the paper they state that they "sampl[ed] fixation lengths from the empirical fixation data...."

Response 14: Thank you, that's our mistake. We've changed the main text:

"Krajbich et al. used randomly sampled fixation durations from the empirical distribution to perform the simulation both in this and later works."

(15) I think it would be more accurate to state that there is "some" evidence for collapsing bounds. Milosvaljevic et al. 2010 argue that collapsing bounds do not help once across trial drift variability is accounted for. Hawkins et al. 2015 show mixed evidence for collapsing bounds. And here, while collapsing bounds do seem to help in the goodness-of-fits statistics, qualitatively the improvement is imperceptible, at least in the plots the authors have shown.

Response 15: That's a fair point. We modified our text in the discussion session as follows:

"...thus we provided some supports for collapsing bound as a decision model components (although, evidence is mixed in the literature (Milosvaljevic et al. 2010, Hawkins et al. 2015))."

(16) Prospect theory states that risk-seeking occurs in the loss domain because of diminishing marginal sensitivity, not "so that they will still have some probability to obtain a good outcome." So while this is an interesting way to think about the loss domain, it is not based on the standard interpretation.

Response 16: Thank you. We deleted this part of imprecise interpretation in our discussion.

(17) Table 2 - please briefly describe the models in the table caption.

Response 17:

Thank you, we've added the caption in table 2 (note the table content is different now). The caption now reads as follows:

Comparison between the main PUC model and its variants, in terms of differences in negative log likelihood, AICc, and BIC. Lower means that the main PUC model is better. All values are summed across subjects; bootstrapped 95% confidence intervals are given in parentheses. The first two columns are models with more flexible parameters (prior variance being flexible; both prior variance and mean being flexible); the third is a reduced PUC model which does not have an uncertainty averse term in the utility (i.e. $A = 0$).All PUC model variants fit worse than the main PUC model.

Referee 2's comments: In this work, Li and Ma propose a partial normative model to study the effects of fixation on decision-making. In the model they propose, the utility is computed based on weighted sum of the posterior mean and standard deviation of the posterior distribution. These values depend on both the alternative input values and the amount of time fixating at each alternative. I think that this is an interesting attempt to formalize the originally proposed instantiation of the aDDM. There are however a couple of critical aspects that the authors may want to clarify related key assumptions adopted in their models and parameter fitting.

Major comments 1) The authors adopt a Bayesian approach to estimate a posterior distribution, which ultimately leads to the computation of an utility that is then used to compute the decision variable (by comparing the utilities of each alternative). The authors assume a Gaussian prior and a Gaussian likelihood function with constant noise (irrespective of value input). The authors use the choice data to fit both the prior (mean and variance), and the variance in the likelihood function. Here there are two critical issues. First, given that the authors fit both prior and likelihood using the choice data, there must be at least some evidence that the use of this prior shape (and the resulting fit) is valid. Do the rating data follow a Gaussian distribution? Is the estimated prior distribution comparable to the rating data distribution? It is essential that the authors show and report this information in the article. I appreciate that the authors briefly mention in the discussion section the fact that the Gaussian distribution assumption is a limitation. However, in my opinion, such strong assumptions should be backed up by a principled argument otherwise the Bayesian model ceases to be "normative". Also, the motivation for such assumption should be stated already in the description of the model. This is not to say that I am against Bayesian approaches (actually quite the opposite), but this is precisely the issue that one sector cognitive psychology has used as ammunition against Bayesian approaches in our field. The authors could for instance obtain the best possible fit of a Gaussian distribution to the rating data and then use this as prior distribution to fit the choice data. Alternatively, out of sample fits could be adopted to estimate out of sample priors (however, this might not sully solve the problem).

Response 18: Thank you. In principle we do not fully agree that showing the normality of rating is critical to make our model a useful insight. But we agree it is helpful to check the empirical rating distribution and see is it very far a way from Gaussian. Here we've attached the rating histogram in Fig9 and also a Q-Q plot (Fig 10). In the range of 0-10 (decided by the experiment) it is satisfyingly normal.

We also agree with the suggestion of "obtain the best possible fit of a Gaussian distribution to the rating data and then use this as prior distribution to fit the choice data". In fact, given the normality of rating distribution, we now instead report a simpler PUC model where the prior mean and variance of the model are directly fixed at the empirical mean and variance of each subject. In the "Model fitting and model comparison" section in the main text, we added the following:

The PUC model, as introduced in "Decision models" above, has 4 parameters for the value estimation (σ, σ_p, μ_p,A), three bound parameters (B_0, k, and λ), one lapse rate parameter (l) and a non-decision time parameter τ. To simplify, we fixed the prior mean σ_p and variance μ_p to be the empirical mean and variance extracted from the rating data, thus leaving 5 parameters to be fitted. We tested the more flexible versions of PUC too.

This simplified model, in fact, outperforms the full PUC model under the criteria of AICc and BIC, indicating that the prior components of our PUC model is indeed well approximated by the empirical

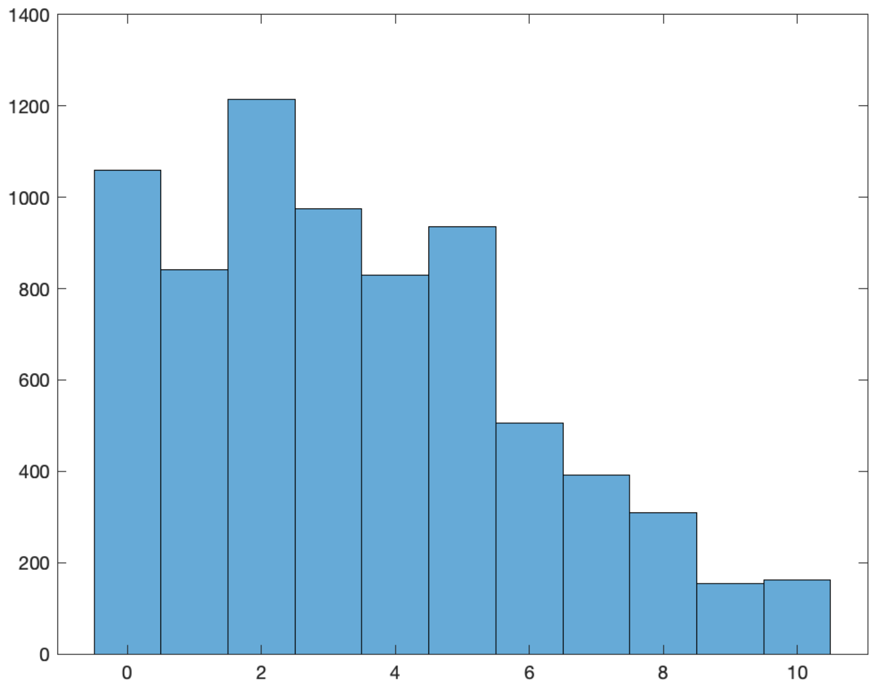

Figure 9: Histogram of rating distribution across all participants.

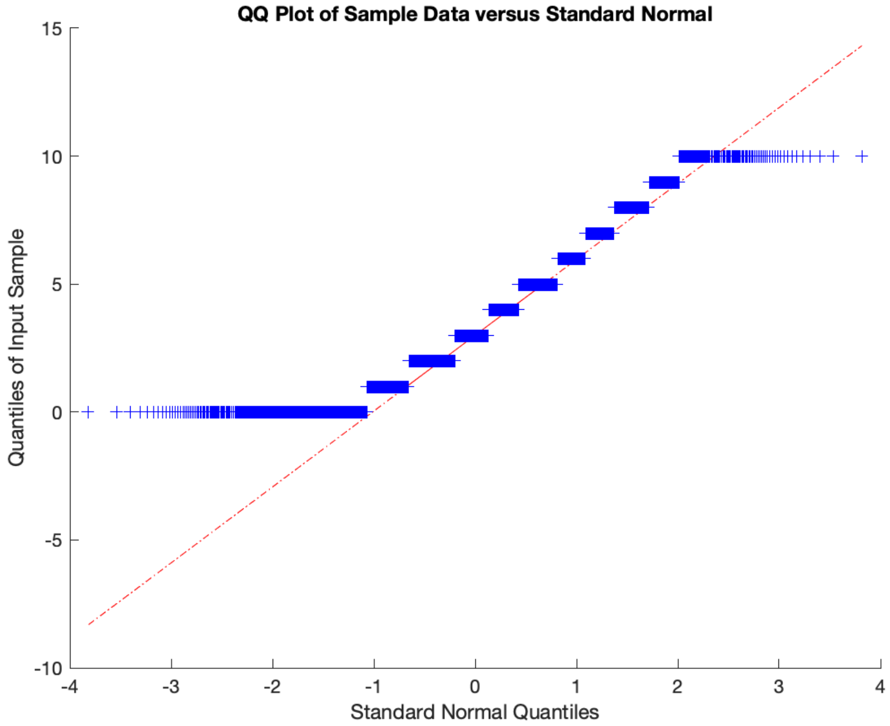

Figure 10: QQ plot of the rating data across all participants.

rating mean. The model comparison is added in the appendix and we attach it here:

	flexible prior var	flexible prior mean & var	uncertainty neutral
neg LL	19 (34, 9)	49 (71, 33)	-655 (-455, -923)
AICc	-56 (-75, -25)	-92 (-124, -47)	-1219 (-1756, -835)
BIC	-140 (-159, -108)	-256 (-288, -212)	-1133 (-1666, -741)

Table 2: Comparison between the main PUC model and its variants, in terms of differences in negative log likelihood, AICc, and BIC. Lower means that the main PUC model is better. All values are summed across subjects; bootstrapped 95% confidence intervals are given in parentheses. The first two columns represent models with more free parameters (prior variance, or both prior mean and prior variance); the third represents a reduced version of the PUC model that does not have an uncertainty-averse term in the utility (i.e. $A = 0$). All PUC model variants fit worse than the main PUC model.

2) The model proposed by the authors has 8 free parameters that are used to fit about 95 choice trials per participant. It has been previously argued that the reliability of fitting that many parameters to DDMs (in particular with dynamic bounds) can be problematic and might be not identifiable, in particular for low number of trials as is the case here. A more critical problem is that the authors are also freely fitting the prior distribution. As it has been shown in previous work, some DDM parameters and in particular boundary parameters are very much related to prior distributions (see related work by Drugowitsch). This in addition to the prior fitting issue highlighted in my previous point 1. Here it is essential to demonstrate via simulations that the Bayesian inference parameters and the boundary parameters are identifiable using the same number of trials used to fit the real data (i.e., about 100 trials). Without this evidence, it is difficult to evaluate whether all free model parameters assumed by the authors are jointly identifiable.

Response 19: Thank you for highlighting the concerns regarding large number of free parameters.

To address this issue, the first major change we have made was the simplified model as mentioned in the response above.

We also generated synthetic data by using the exact same rating distribution as the real data, fitted the synthetic data with the same procedure as in fitting real data (using Bayesian Adaptive Direct Search to find the fitted parameters), then compared the optimal parameter and the resulting model predictions with the ground truth. In Fig11, we confirmed that the summary statistics can be near perfectly recovered with all the models, as an intuitive check of the validity of our model fitting procedure.

We performed parameter recovery for all three models, while fixing the non-decision time and lapse rate to their generating values. Thus, we are left with 3 parameters for aDDM and 5 for both acbDDM and the modified PUC model. Parameter recovery is good for the aDDM (Fig 12), and less good for the more complex acbDDM and PUC models, with some parameters being recovered well and some less well (Fig 13, Fig 14). This is likely due to trade-offs between parameters, where a change in one parameter can be compensated for by changes in one or more other parameters, to produce an approximately equally high log likelihood. Importantly, however, the results of our paper do not rely on parameter estimates but only on maximal log likelihoods (and AICc/BIC), so issues with parameter recovery will not affect our conclusion. That being said, we agree with the reviewer that questions about the reliability of the parameter estimates are important.

We have now incorporated these parameter recovery results to our main manuscript in the supplement.

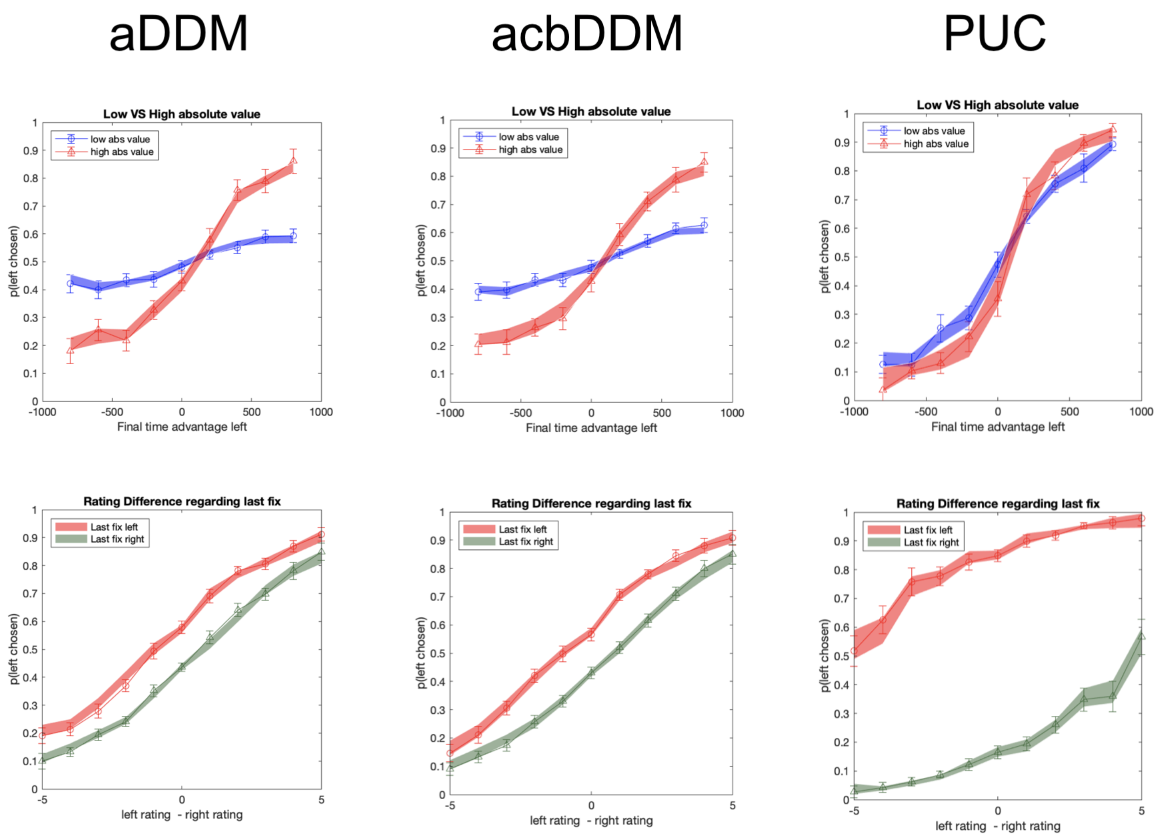

Figure 11: Parameter recovery with synthetic data. The fitted parameters are able to perfectly recover the major summary statistics.

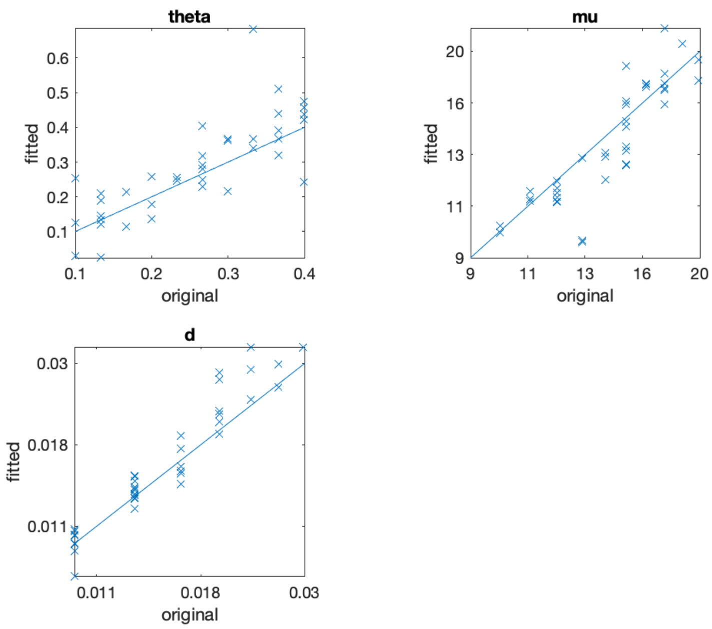

Figure 12: aDDM: Parameter recovery with synthetic data, comparing the parameter distribution. Some parameters are plotted in log space because that is how they are fitted.

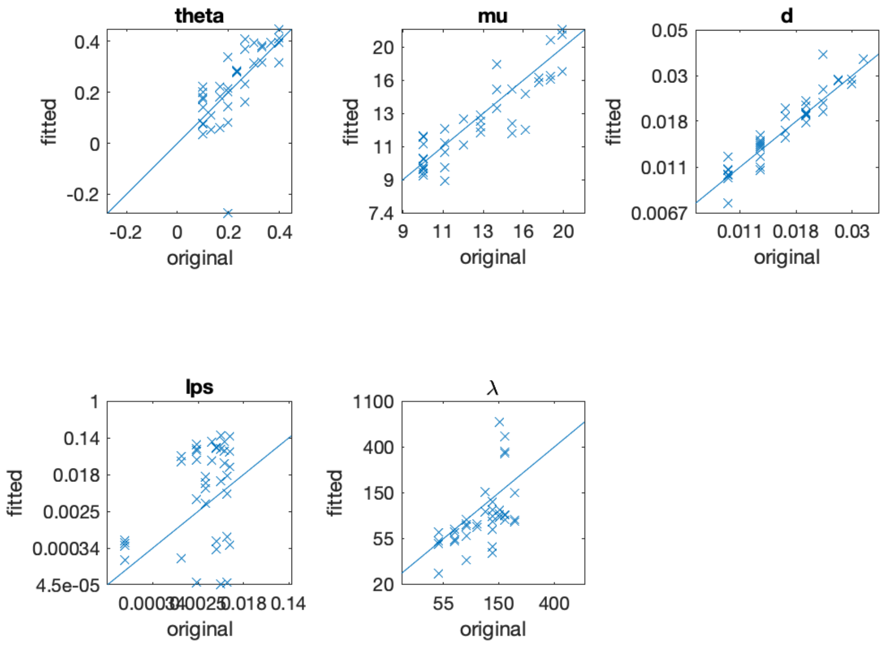

Figure 13: acbDDM: Parameter recovery with synthetic data, comparing the parameter distribution. Some parameters are plotted in log space because that is how they are fitted.

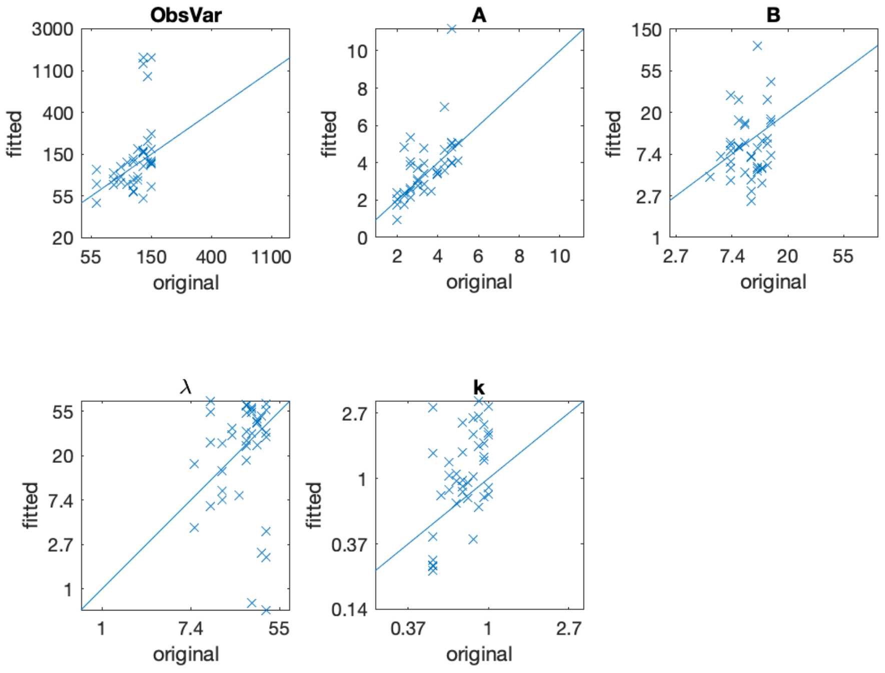

Figure 14: PUC: Parameter recovery with synthetic data, comparing the parameter distribution. Some parameters are plotted in log space because that is how they are fitted.

In the main text we added this method paragraph:

Parameter recovery and model checking. To confirm the validity of our model fitting choices, we fitted synthetic data using the same fitting procedures as for the real data. To generate synthetic data, we used the exact same rating distribution as the real data by matching each synthetic trial with a real trial. Each synthetic subject had different set of parameters randomly chosen from a given range. Then we performed fitting for individual synthetic subjects using methods introduced above. Results are presented in the supplementary material. The summary statistics are recovered very well. Parameter recovery is generally good but somewhat worse for the more complex models (acbDDM and PUC). This is likely due to soft trade-offs between parameters. As a result, the parameter estimates in the real data should be taken with a grain of salt. However, the results of our paper do not rely on parameter estimates but only on log likelihoods and summary statistics. Therefore, the results are not affected by issues with parameter recovery.

Minor comment

3) It would be informative to study further qualitative predictions that could uniquely emerge in the model proposed by the authors. At present it is not clear (at least qualitatively) what the key differences are between the PUC and the aDDM. For instance, it is possible that the model makes different predictions for long versus short trials as in the PUC the model is directly influenced by viewing time? Response 20:

This is a great suggestion! One thing our qualitative model comparison lacks is the comparison regarding how long it takes before making a decision. So now we added this additional summary statistics about the P(make choice) regarding the total fixation time (Fig. 15, fourth column). We found that aDDM predicts the average trial length to be shorter than PUC. We do not argue, however, that this is an intrinsic quality difference between aDDM and PUC because these plots are obtained with the best fitted parameters of each model, i.e. they are mostly constrained by the specific data we are fitting.

We reported this new analysis in Fig. 3 of the updated manuscript and the accompanying text.

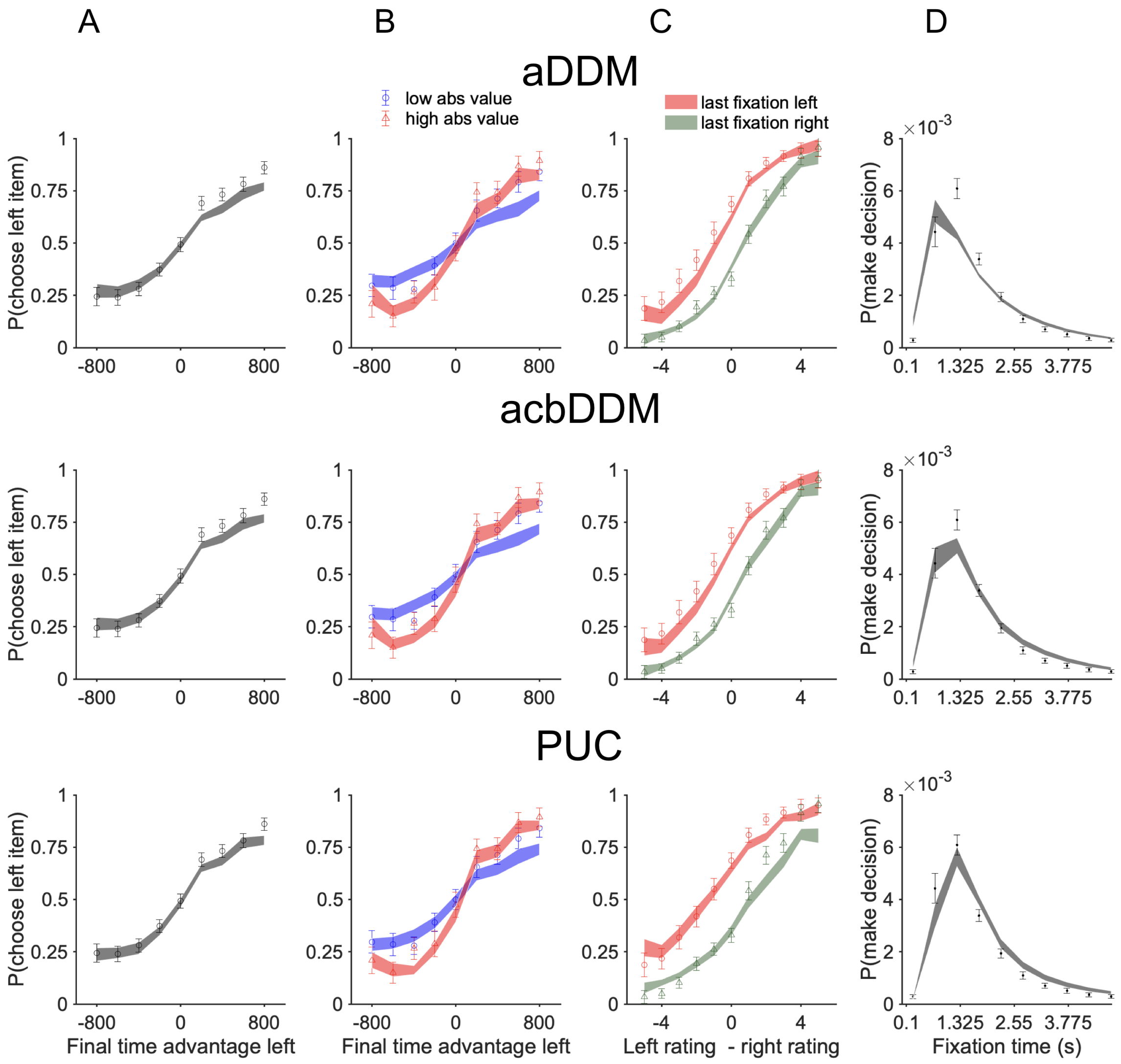

Figure 15: Fits of the aDDM, the acbDDM, and the PUC model to summary statistics of the data. (A) When the total fixation time advantage of an item increases, that item is chosen more often. (B) Same as A but conditioned on item rating. Both models predicted that when the absolute values of both items are higher (i.e. both are more preferred items), the fixation modulation effect is larger, which trend is less significant in the empirical data. (C) Besides total fixation duration, the last fixation also biases the choice. (D) Distribution of total fixation time. Note for this figure we simulated fixation series extending after the empirical total fixation time. The aDDM fits we obtained differ from those in Krajbich et al.(2010) not only because of the differences in parameter estimation methods (see section "Differences from Krajbich et al. (2010)."), but also because of a difference in trial aggregation: Krajbich et al. aggregated the model predictions across rating pairs without taking into account the frequencies of these pairs in the experiment. We instead aggregated individual-trial predictions; this is necessary in our case because we used the individual-trial fixation series, but it also ensures a proportional representation of each rating pair in the summary statistics.
