# Supplementary material for: An uncertainty-based model of the effects of fixation on choice
Source: PLoS Comput Biol. 2021 Aug 16;17(8):e1009190. doi: 10.1371/journal.pcbi.1009190 (PMC8389845; doi:10.1371/journal.pcbi.1009190)
Supplement: Attachment — Submitted filename: second_response_to_review_fixation.pdf [file pcbi.1009190.s005.pdf]

**Zhi-Wei Li**
6 Washington Place
New York University
New York, NY, 10003
Email: zhiwei.li@nyu.edu

**June 7, 2021**

Dear editors,

We would like to thank Reviewer 1 for their remaining comments, and Reviewer 2 for their positive judgments. We have addressed Reviewer's comments point by point below. Our responses to the comments are numbered and marked in **blue**. References to updated passages in the manuscript are marked in **green**.

Sincerely,

Zhi-Wei Li and Wei Ji Ma

**Referee 1's comments**: (1) One of the takeaway points of the paper is that collapsing bounds are important, as they improve the aDDM fits substantially. However, one issue with this claim is that the boundary parameters k and lambda were constrained to be positive in the fits, and there appears to be bunching of the estimates close to or at zero. This constraint essentially guarantees that you will find support for collapsing bounds as long as there is variability across participants. I'm not asking the authors to refit all the models, but if they don't, I think they need to qualify that they did not allow for increasing bounds over time. What they have shown is that allowing bounds to vary improves model fit, but not that bounds are necessarily collapsing.

Response 1: Thank you, we have emphasized this inflexibility of our model in the section of "aDDM with collapsing bounds" by adding the following clarification:

In principle, we could also allow for an increasing bound by changing the parameter ranges of the boundary function. We limit ourselves to a collapsing bound here, in view of previous work and because it is psychologically easier to interpret.

We also edited our comments regarding this model advantage in our discussion section:

Thus we provided some supports for a flexible bound as a decision model component (although, evidence is mixed in the literature, see...).

(2) I think the authors need to be clearer about the parameter recovery results in the main text. I wouldn't say that parameter recovery is "somewhat worse" for the more complex models; it is non-existent. To my eye the correlation between true and fitted parameters looks to be about 0 for several of the parameters, for example lambda and k in the PUC model. While this might not impact model comparison, it does mean that these parameters can't really be interpreted, so readers should not try to fit these models to data and compare parameter values (as readers are wont to do).

Side note - where do the "original" parameter values come from anyways? They don't appear to be from the participants' best fitting values.

Response 2: It is a great point that the "original" parameters should come from the participants' best fitting values. This should help to avoid potentially nonsensical data produced by arbitrary parameters. We changed the parameter recovery analysis by using the participants' best fitting values. The parameter recovery results are much better, see Fig. 1 below; this figure is also in the supplement.

The new procedure have been updated in the main text:

Each synthetic subject was given the parameters that best fitted one real subject.

(3) In the "Differences between PUC and aDDM" section, point (c) says that in the aDDM the information about the two items is combined into a single variable while in the PUC there are two distinct value distributions. However, Krajbich & Rangel 2011 showed that the multi-alternative version of aDDM has separate accumulators for each item and that it reduces to the aDDM with two items. In other words, the aDDM can be thought of as two distinct accumulators with a downstream difference calculation to compute the relative variable. In other words, both models allow the agent to keep two distinct value representations, and in the multi-alternative case this is required. I would suggest removing point (c).

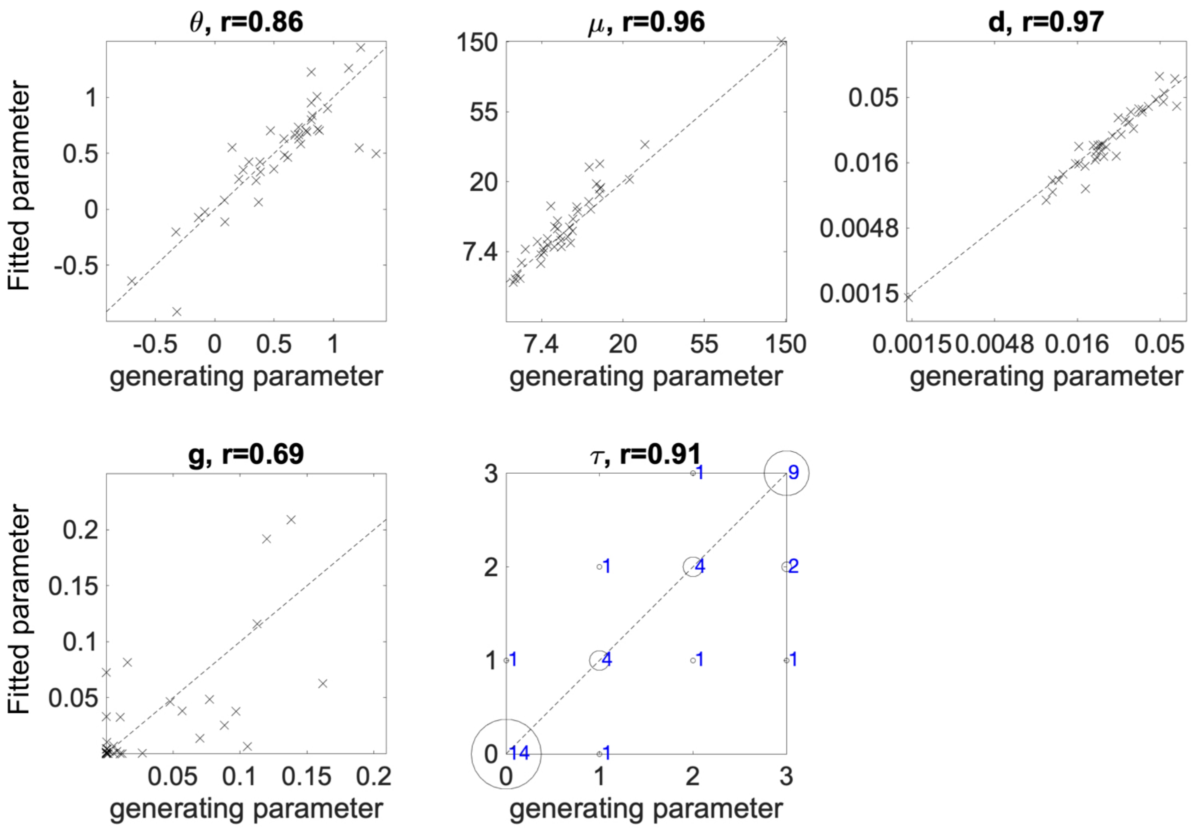


**Figure 1:** Parameter recovery in the aDDM. We generated and then fitted synthetic data sets using the parameter estimates of the real subjects (for a total of 39 data sets). Shown are the fitted parameters as a function of the generating parameters, with the Pearson correlation given in the plot title. Some parameters are plotted in log space because that is how they were fitted; the Pearson correlation is then also calculated for the log parameter. For the non-decision time parameter $\tau$, which has discrete values, circle circumference is proportional to the number of data points as annotated.

Response 3: Thank you, we removed this point from the list and instead added at the end:

"In addition, on the surface, it seems that in the PUC model, the agent keeps two distinct value distributions, one for each item, whereas in the a(cb)DDM, the information of the two items are combined into a single "relative decision variable". However, in the later extension of aDDM model where more than two options are being compared (Krajbich & Rangel 2011), separate accumulators for each item were used and and it could be reduced to the aDDM with two items. Thus, the a(cb)DDM can also be conceptualized as two distinct accumulators, followed by another step of deriving the relative value difference, which is similar to the PUC model."

Minor comments What are the units on d? I guess this is in "evidence per 100ms"?

Response 4: $d$ is in unit of "time step" or "100ms" in our case; Related, the unit of $\sigma$ is "squared time step".

aDDM B set to 0? I think you mean 1?

Response 5: Thank you, that was a typo and we have fixed it.

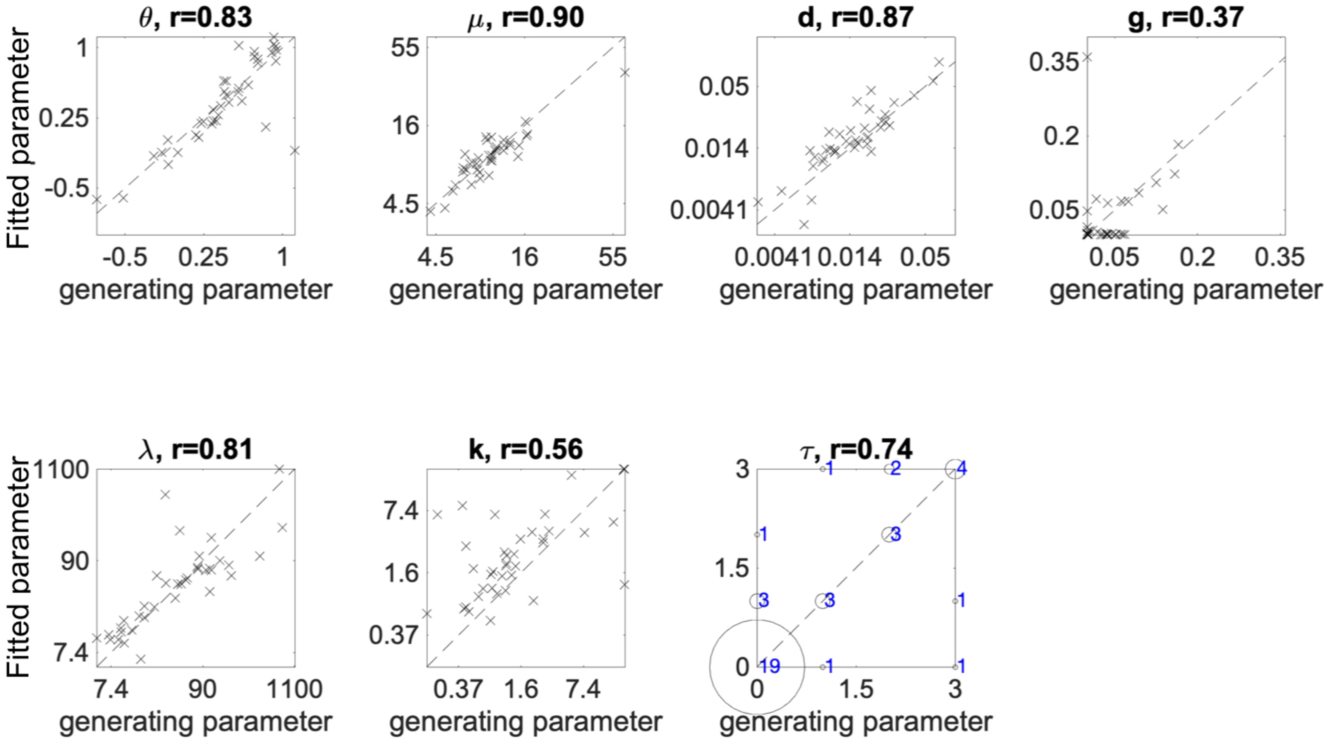


**Figure 2:** Parameter recovery in the acbDDM. For details, see Figure 1.

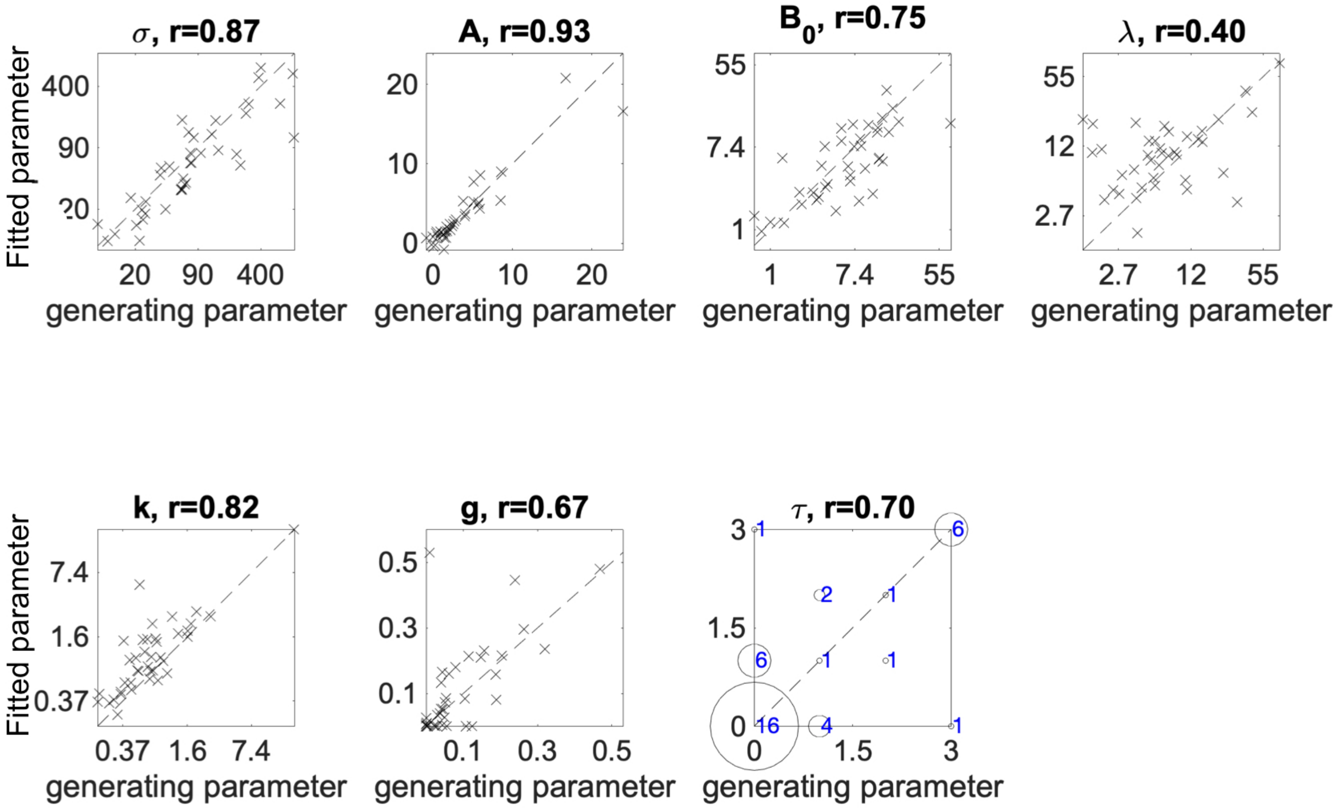


**Figure 3:** Parameter recovery in the PUC model. For details, see Figure 1.

The PUC has 9 parameters, you fix 2, but that leaves 7 not 5...

Response 6: Thank you, that was a typo and has been fixed.

The "DDM2 parameter recovery" figure has one panel that says "lps" with unreadable units - should that be k?

Response 7: That should have been the "lapse rate". For better readability, we have changed "lapse rate" to "guessing rate", abbreviated with "$g$".

The PUC parameter recovery figure has a panel labeled "ObsVar" - should that be sigma?

Response 8: Yes we have changed that to be $\sigma$. Thanks for pointing that out.

Across the parameter recovery and parameter distribution figures, you are inconsistent in whether you use the greek symbol itself or spell it out.

Response 9: Thank you. We have updated the parameter recovery figure to make it consistent with the parameter distribution figures. We also corrected "ndT" to be "$\tau$" and changed "lapse rate" to "guessing rate", abbreviated with "$g$", for better readability.
